# Directionality and representativeness are differentiable components of stereotypes in large language models

**DOI:** 10.1093/pnasnexus/pgae493

**Published:** 2024-11-04

**Authors:** Gandalf Nicolas, Aylin Caliskan

**Affiliations:** Department of Psychology, Rutgers University, New Brunswick, NJ 08873, USA; The Information School, University of Washington, Seattle, WA 98195, USA

**Keywords:** bias in Artificial Intelligence, stereotypes, fairness in machine learning, natural language processing, social psychology

## Abstract

Representativeness is a relevant but unexamined property of stereotypes in language models. Existing auditing and debiasing approaches address the direction of stereotypes, such as whether a social category (e.g. men, women) is associated more with incompetence vs. competence content. On the other hand, representativeness is the extent to which a social category's stereotypes are about a specific content dimension, such as Competence, regardless of direction (e.g. as indicated by how often dimension-related words appear in stereotypes about the social category). As such, two social categories may be associated with competence (vs. incompetence), yet one category's stereotypes are mostly about competence, whereas the other's are mostly about alternative content (e.g. Warmth). Such differentiability would suggest that direction-based auditing may fail to identify biases in content representativeness. Here, we use a large sample of social categories that are salient in American society (based on gender, race, occupation, and others) to examine whether representativeness is an independent feature of stereotypes in the ChatGPT chatbot and SBERT language model. We focus on the Warmth and Competence stereotype dimensions, given their well-established centrality in human stereotype content. Our results provide evidence for the construct differentiability of direction and representativeness for Warmth and Competence stereotypes across models and target stimuli (social category terms, racialized name exemplars). Additionally, both direction and representativeness uniquely predicted the models' internal general valence (positivity vs. negativity) and human stereotypes. We discuss implications for the use of AI in the study of human cognition and the field of fairness in AI.

**Significance Statement**

Representativeness is the extent to which stereotypes about a social category (e.g. men, women) are about a specific content dimension (e.g. Warmth or Competence). Direction refers to where in the dimension do these stereotypes fall (from low to high; e.g. unfriendly to friendly for Warmth). For example, if women are stereotyped as “smart,” “assertive,” and “warm,” then Competence is more representative than Warmth (2 vs. 1 traits), but direction is high for both dimensions. In the ChatGPT and SBERT language models, representativeness is differentiable from direction and improves prediction of human and language model evaluations of categories' valence (positivity/negativity). Incorporating both representativeness and direction measures would improve inferences about human cultural patterns and help identify unaddressed biases in language models.

## Directionality and representativeness are differentiable components of stereotypes in language models

A deeper understanding of the various ways in which stereotypes manifest will improve bias detection and mitigation for artificial intelligence (AI) models. In the current paper, we examine whether language models' associations with social categories show differentiable biases for directionality and representativeness, reproducing patterns in human studies (1).

Multiple social categories are salient in any given society (e.g. gender, age, race, occupation (2)). People hold beliefs about the characteristics of the members of a social category (i.e. stereotypes (3)). These stereotypes may be inaccurate^a^, over-generalized, or self-fulfilling, among other well-documented issues (5), often resulting in discrimination, conflict, and decreased health of stigmatized groups (6, 7).

### Stereotype direction

The well-established primary dimensions of stereotype content are Warmth (also called the horizontal dimension, or Communion) and Competence (also called the vertical dimension, or Agency), referring respectively to beliefs about a target's perceived moral character and friendliness, and perceived abilities and assertiveness (3). Dimensions vary in directionality: how low to high the characteristic is, along a semantic differential scale. For example, a target could be evaluated as low Warmth (e.g. “unfriendly,” “insincere”) or high Warmth (e.g. “friendly,” “sincere”). As such, social categories can be stereotyped as high on both Warmth and Competence (e.g. Americans), low on both (e.g. people who are homeless), high Warmth but low Competence, (e.g. children), or low Warmth but high Competence (e.g. people who are rich). Warmth and Competence direction predict many outcomes, from social emotions to behavioral intentions and decision-making (6, 8, 9).

Direction is related to valence (i.e. the positivity/negativity of the stereotype): high Warmth or Competence words (e.g. “amicable” or “smart”) tend to be positively valenced, while low words (e.g. “enemy” or “unintelligent”) tend to be negative^b^. Valence may also be measured more generally, collapsing across specific dimensions of content such as Warmth and Competence. This “general valence” is a measure of attitude or prejudice toward the category (1). Both Warmth and Competence direction predict general valence, with Warmth direction being particularly predictive (8). However, measures of valence and direction do not capture additional stereotype properties, such as associative strength with the content dimension.

### Stereotype representativeness

Stereotypes can be theorized as part of an associative network in memory, where characteristics and social categories are interconnected nodes, and where these connections may vary in strength (9). In this framework, how much a stereotype is activated upon encountering the social category partially depends on the strength of the association in memory (10). Here, we focus on stereotype representativeness, a measure of associative strength with a content dimension with direct applicability to language models.

The spontaneous stereotype content model (SSCM (1)) is a recent model that integrates text analysis and survey free response measures to differentiate direction from representativeness. The SSCM identified the construct of “content representativeness” as the prevalence of a content dimension in stereotypes about a category. That is, a stereotype dimension, such as Competence, is more representative of a social category if the category is often associated with Competence-related content, including stereotypes such as “smart,” “educated,” and “unassertive” (note that the direction/valence of the stereotypes does not matter for this metric, as long as they are about the dimension). The category would have the lowest representativeness for Warmth if none of its stereotypes are about Warmth-related traits.

To further illustrate, when asked to rate stereotype direction for nurses and doctors using numerical scales (e.g. 1—Not at all warm to 5—Very warm), American participants provide similarly high Warmth and Competence direction evaluations of both. However, when freely listing the characteristics they associate with the categories, using text responses, Americans mostly use Warmth-related words for nurses and Competence-related words for doctors. That is, Warmth is a more representative stereotype dimension of the nurse category, while Competence is more representative of the doctor category. Similar patterns appear for other categories: direction ratings of Black and Asian targets are similarly neutral on both dimensions, but Warmth is more representative of stereotypes of Black targets and Competence is more representative of Asian targets (1).

This pattern, where categories may be rated as similar along a dimension despite differences in how much the dimension comes spontaneously to mind (vs. other content dimensions), is evidence of two distinct constructs: direction and representativeness. Moreover, representativeness and direction have an interactive effect on general evaluations of a target and in decision-making scenarios about social categories. For example, a dimension's direction better predicts general valence evaluations of a target when the dimension is more representative (1). Thus, understanding both direction and representativeness is necessary for a more comprehensive account of bias.

### Stereotype research in AI

Language models have become ubiquitous in applications with real-world impact, from healthcare (11) to hiring (12). However, these models, trained on human data, reflect and perpetuate societal biases (13).

Most research on stereotypes in language models has focused on biases in general valence using text embeddings, since this is an easy-to-measure construct with important implications (e.g. 14–17). Embeddings are numerical representations of the semantic relations between words, obtained through language models. These numerical representations allow for the placement of words in a multidimensional space, such that more semantically and contextually related words are closer together in this space. The model learns and creates the embeddings by observing word co-occurrences and regularities in the vast natural language training data (e.g. Google Books, a crawl of the internet). In this way, the position of words in the multidimensional space will capture stereotypical associations between social category terms (e.g. young, Muslim, woman) and various positive and negative traits (14). We note that these models focus on English in an American context, given their training data (18), and so here we focus on stereotypes in the United States.

Correlations between text embeddings show that language models capture general valence biases about multiple categories, including age, race, and gender (e.g. 19, 20). Embeddings can also provide “top words” associated with social categories, which are then coded using additional methods (e.g. dictionaries) to measure valence bias (17). Recently, researchers have begun to examine dimensionality beyond general valence. For example, studies have looked at gender–concept associations and identified multiple domains of content, including specific dimensions of valence–potency–activity (16), which partially overlap with Warmth and Competence (21). Other research has begun to examine directionality along Warmth and Competence dimensions in language models (22–25).

## Current study

Research on AI bias has focused on valence/directional associations without examining strength of association with the content dimension. In fact, many debiasing methods rely on directionality correction, for example, using antistereotypes (24, 25). We argue that such an approach fails to identify (and potentially correct) biases based on the representativeness of content.

Because representativeness is a property of human stereotypes (1), higher correspondence between human and AI stereotypes may also be achieved by incorporating measures of this property in AI studies. This would improve integration of findings using AI models with psychological theories and help us quantify how well auditing methods are capturing known human stereotypes. Furthermore, because in human data both representativeness and direction independently relate to attitudes toward social targets (1), examining both properties in AI may reveal information about a language model's overall biases toward social categories.

In the current study, we introduce the construct of content representativeness to the study of bias in AI. We examine whether representativeness and direction are differentiable stereotype properties (i.e. our differentiability hypothesis) by examining how much variance they share. Furthermore, we illustrate the expected differentiation through specific social categories with varying direction and representativeness. Finally, we hypothesize that considering representativeness in addition to direction will improve correlations with the models' and human general valence measures.

In addition to shedding light on the role of representativeness, the current study explores variation in bias as a function of the model, method of measurement, and target type. Specifically, we use two of the latest and most powerful language models: GPT 3.5 turbo, as implemented in the widely used ChatGPT chatbot, and Sentence Bert (SBERT). Both involve contextualized text representations (i.e. embeddings that account for contextual information in a sentence). However, they also facilitate different quantitative approaches: for ChatGPT, we focus on a “conversational” method, where the model is prompted in plain language to provide a list of characteristics culturally associated with each social category, whereas for SBERT we make direct use of the text embeddings to retrieve top word associations with each social category (17). Associations for both approaches are then coded using validated dictionaries to obtain both direction and representativeness scores (1). In addition, we obtain general valence scores (i.e. how negatively to positively is the category evaluated, on a five-point scale) from both the language models themselves and from human participants, to understand their relationship with the representativeness and direction constructs.

Most of the research in the field has focused on category-specific associations, often by using gendered and racialized names. Recent exceptions (17, 26, 27) have incorporated large samples of social category terms to examine patterns across categories. However, no comparison between these approaches has been made, opening the question of whether they capture biases differently. Here, we focus on examining terms for multiple salient social categories given its more general nature, but use both approaches, allowing us to explore whether different biases arise based on target type (i.e. category terms vs. racialized name exemplars).

Our multimethod and multimodel approach aims primarily to establish the robustness of the differentiability hypothesis. Secondarily, it will allow us to explore variation based on the model, training data, targets, and/or methodology (but additional studies will be needed for more controlled comparisons).

## Results

### Variance explained

As a test of the differentiability of direction and representativeness, we measured how much of the representativeness variance was accounted for by the direction indicators for the corresponding dimension. In line with our differentiability hypothesis, a quadratic model for Warmth direction accounted for a minority of the variance in Warmth representativeness (ChatGPT: *R*^2^ = 0.051; SBERT: *R*^2^ = 0.038). A similar model for Competence direction also accounted for a small amount of Competence representativeness variance (ChatGPT: *R*^2^ = 0.063; SBERT: *R*^2^ = 0.033). Thus, although direction and extremity (i.e. direction squared) correlate with representativeness, they account for a minority of the variance in this variable, indicating differentiability.

### Across-category differentiation

**Fig. 1:**
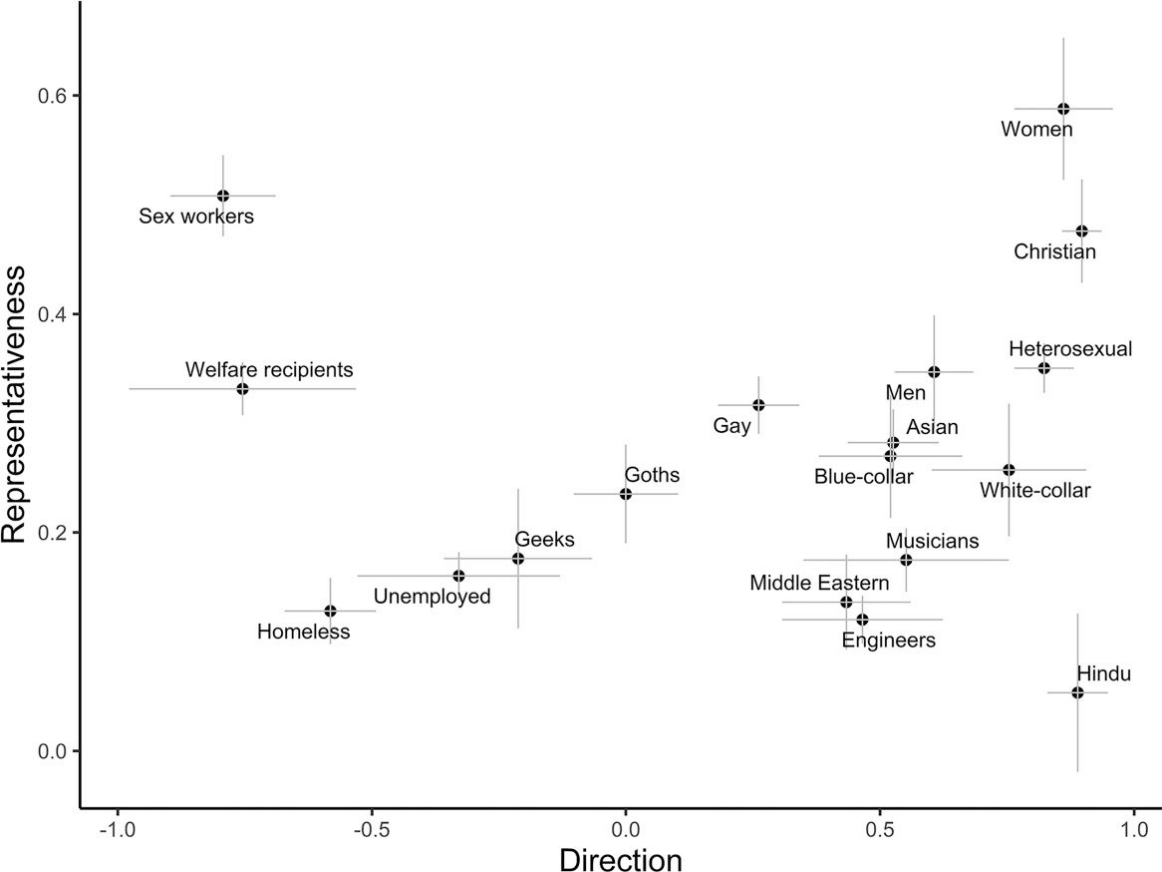
Warmth direction and representativeness for category terms, ChatGPT. Direction ranges from −1, low (e.g. immoral) to 1, high (e.g. moral) Warmth, while representative ranges from 0 to 1, with values indicating the proportion of responses that were about Warmth (regardless of direction). Only a subset of the full list of categories shown. The error bars are ±1 SEs.

**Fig. 2.**
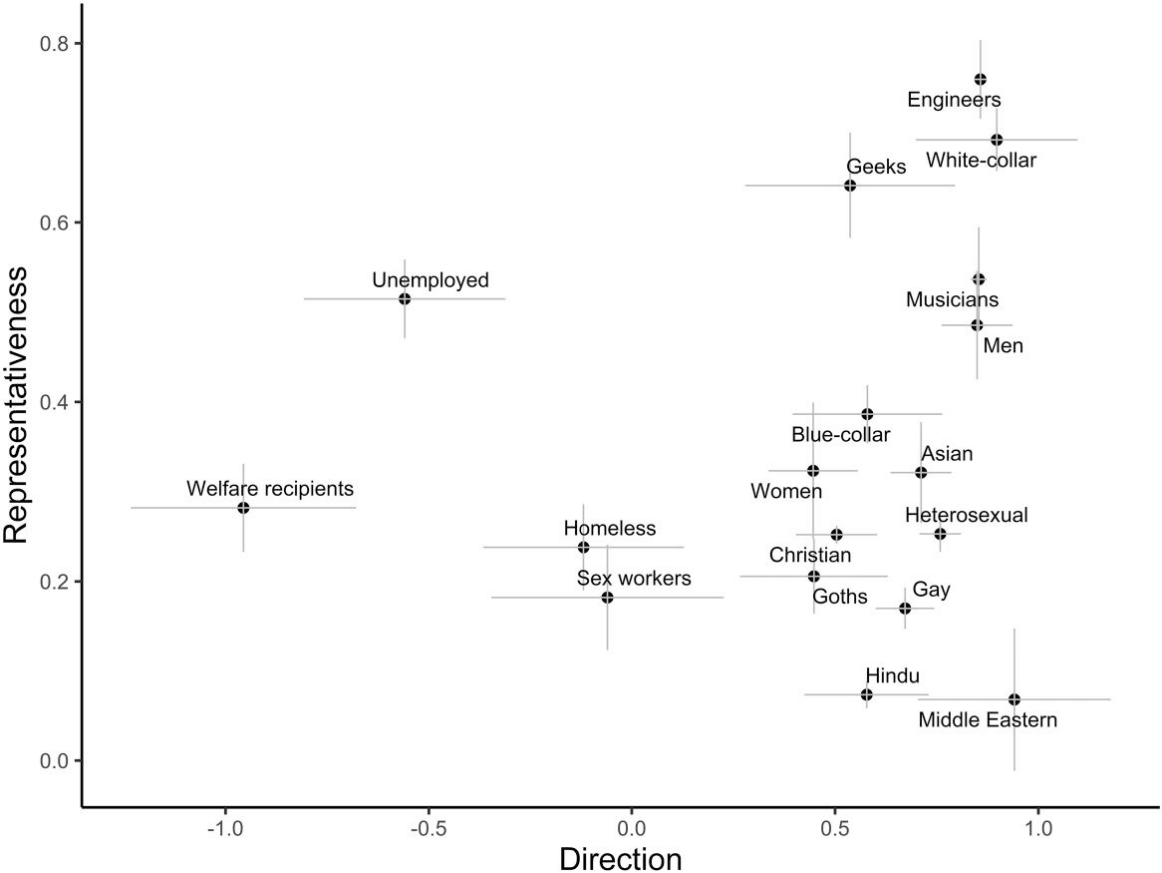
Competence direction and representativeness for category terms, ChatGPT. Direction ranges from −1, low (e.g. unintelligent) to 1, high (e.g. intelligent) Competence, while representative ranges from 0 to 1, with values indicating the proportion of responses that were about Competence (regardless of direction). Only a subset of the full list of categories shown. The error bars are ±1 SEs.

The direction–representativeness differentiability can also be seen in the distribution of categories along these measures. In Figs. 1 and 2 (using ChatGPT), we illustrate how multiple categories show similar direction scores, yet dissimilarity on representativeness. For example, for Warmth, “welfare recipients” and “unemployed” have relatively negative direction scores that are not significantly different (Cohen's *d* = 0.96, *P* = 0.197), but this negative Warmth is more strongly associated with welfare recipients (vs. unemployed people; *d* = 3.55, *P* < 0.001). As additional examples, direction scores for “Christian” and “Hindu” categories (*d* = 0.08, *P* = 0.915), and for “men” and “women” (*d* = 1.00, *P* = 0.056), are similar and not significantly different, yet Warmth is more representative of associations with Christians (vs. Hindus; *d* = 3.38, *P* = 0.001) and women (vs. men; *d* = 1.40, *P* = 0.010). Similar patterns are evident for Competence. For example, “engineer” and “musician” categories have similarly positive direction (*d* = 0.07, *P* = 0.875), but more Competence words associate with “engineer” than with “musician” (*d* = 1.36, *P* = 0.006); “blue-collar” and “white-collar” occupation categories have similar direction (*d* = 0.65, *P* = 0.265), but Competence is more representative of “white-collar” (*d* = 3.57, *P* < 0.001). Direction–representativeness differentiability occurs for multiple other categories, including “geeks” and “goths” (for Competence), “gay” and “heterosexual” (for Competence), “sex workers” and “homeless” (for Warmth), and “Asian” and “Middle Eastern” (for both Warmth and Competence), among others. SBERT also shows extensive evidence of direction–representativeness differentiability; however, the specific pattern and categories involved may differ, suggesting cross-model variability in specific biases (see Supplement).

### Replications with surname stimuli

Next, we employ more traditional approaches which rely on exemplar items as category signals. Specifically, we used surnames culturally associated with Hispanic, Asian, and White racial categories (in the United States). As with the category terms, these results showed that representativeness was not reducible to quadratic models of direction (although ChatGPT correlations were higher than when using category terms), for both Warmth (ChatGPT: *R*^2^ = 0.366; SBERT: *R*^2^ = 0.045) and Competence (ChatGPT: *R*^2^ = 0.265; SBERT: *R*^2^ = 0.121).

**Fig. 3.**
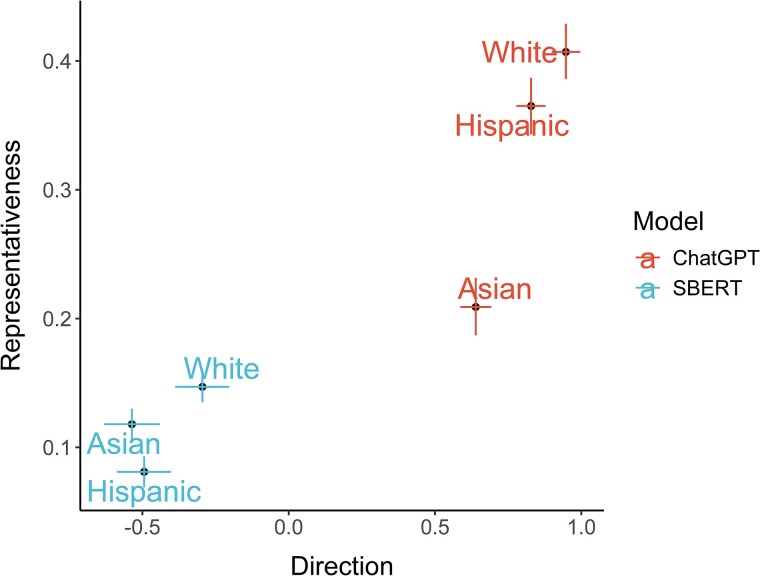
Warmth direction and representativeness for surname exemplars. Direction ranges from −1, low (e.g. unfriendliness) to 1, high (e.g. friendliness) Warmth, while representative ranges from 0 to 1, with higher values indicating that the content dimension is more strongly associated with the group. The error bars are ±1 SEs.

Specific pairwise comparisons are presented in Table 1 and again illustrate differentiability of direction and representativeness. For example, in both language models we found that, in terms of Competence, the direction scores for Hispanic and White surnames were more similar (*d*s < 0.551, *p*s > 0.137) than their representative scores (*d*s > 0.99, *p*s < 0.005). In other words, while traditional direction associations failed to reveal large and statistically significant bias along the Competence dimension, bias was present at the level of representativeness. Instead, Hispanic surnames may be more strongly associated with other content (e.g. geography/foreign stereotypes (1)). For Warmth, similar patterns are found for Hispanic vs. White in SBERT, also shown in Fig. 3. We note that patterns present at the level of racialized surname exemplars did not necessarily occur for category terms, highlighting the importance of examining bias across both more comprehensive high-level terms and lower-level within-category exemplars.

**Table 1.** Representativeness–direction differentiability using racialized surnames in ChatGPT and SBERT.

AI Model	Category	Warmth		Competence	
		Direction	Representativeness	Direction	Representativeness
ChatGPT	Asian	0.639^a^	0.21^a^	0.776^a^	0.34^ab^
	Hispanic	0.828^b^	0.36^b^	0.928^b^	0.31^a^
	White	0.947^b^	0.41^b^	0.884^b^	0.43^b^
SBERT	Asian	−0.295^a^	0.118^ab^	0.522^a^	0.040^ab^
	Hispanic	−0.494^a^	0.081^a^	0.442^a^	0.027^a^
	White	−0.536^a^	0.147^b^	0.064^a^	0.066^b^

Within AI model, for each column, values with the same superscript are not significantly different (*P* > 0.05).

### Predicting internal general valence

To further understand the impact of directionality and representativeness, we can examine how these variables correlate with the language models' internal general measures of valence (using category terms as stimuli). Table 2 shows how representativeness, both as an additive and interactive predictor, adds information beyond that provided by direction, for both Warmth and Competence.

**Table 2.** Nested models showing incremental validity of Warmth and Competence representativeness and its interaction with direction, for ChatGPT and SBERT, in predictions of internal valence.

Model	#	Predictor	*b*	*t*	*P*	*R* ^2^	AIC	*χ*2	*P*
ChatGPT	**1**	**Warmth direction**	0.407	7.68	<0.001				
		**Competence direction**	0.193	2.85	0.005	0.141	1204.14		
	**2**	Warmth direction	0.408	7.78	<0.001				
		Competence direction	0.152	2.27	0.023				
		**Warmth representativeness**	−0.396	−2.23	0.026				
		**Competence representativeness**	0.640	4.23	<0.001	0.203	1179.00	29.14	<0.001
	**3**	Warmth direction	−0.079	−0.57	0.302				
		Competence direction	−0.032	−0.37	0.745				
		Warmth representativeness	−0.448	−2.93	0.008				
		Competence representativeness	0.255	1.22	0.177				
		**Warmth interaction**	1.700	8.17	<0.001				
		**Competence interaction**	0.599	2.71	0.009	0.316	1113.87	69.13	<0.001
SBERT	**1**	**Warmth direction**	0.033	9.25	<0.001				
		**Competence direction**	0.026	7.59	<0.001	0.113	−2765.83		
	**2**	Warmth direction	0.028	9.411	<0.001				
		Competence direction	0.025	8.010	<0.001				
		**Warmth representativeness**	−0.108	−6.076	<0.001				
		**Competence representativeness**	0.042	2.004	0.045	0.161	−2802.37	40.54	<0.001
	**3**	Warmth direction	−0.008	−1.99	0.046				
		Competence direction	0.003	0.83	0.407				
		Warmth representativeness	0.006	0.32	0.752				
		Competence representativeness	−0.093	−3.95	<0.001				
		**Warmth interaction**	0.313	13.62	<0.001				
		**Competence interaction**	0.280	8.72	<0.001	0.337	−3040.00	241.63	<0.001

Model comparison metrics are provided, comparing the three levels of nested regressions for each language model. Higher values for the outcome indicate more positive language model ratings of the social categories.

Specifically, in an initial comparison, a model adding representativeness measures outperformed a model including only direction for internal general valence prediction. The patterns suggest that higher Warmth representativeness predicts more negative internal evaluations of the category, while higher Competence representativeness predicts more positive internal evaluations, when controlling for the direction of the dimension. This is potentially due to Warmth stereotypes being more negative than Competence and other content dimensions (1). A subsequent comparison suggests that a model including interactions between direction and representativeness for each dimension further improved congruence with the models' internal valence associations.

Examining the significant interactions, results are similar to the pattern found in human studies (1): the higher the representativeness of a dimension, the more predictive direction on that dimension is of general valence. To illustrate, in ChatGPT, when Competence is less representative (−1 SD) of the category, whether the target was high or low on Competence direction did not predict general valence toward the target (*b* = 0.064, *P* = 0.384). However, direction was predictive of valence when Competence was more representative (+1 SD; *b* = 0.345, *P* < 0.001). This pattern was present for both Warmth and Competence, across both ChatGPT and SBERT. Results suggest that considering both representativeness and direction improves congruence with language models' internal evaluations of social categories.

### Language models–human correlations

We have presented how, within language models, representativeness and direction disassociate, as they do in human data. But are these variables representing social categories similarly to how humans represent them? As shown in Table 3, codings for language models' Warmth and Competence significantly correlated with their corresponding codings of human stereotypes (convergent correlations). For example, the correlation between Competence direction in ChatGPT responses and human responses was *r* = 0.501. On the other hand, correlations between variables measuring constructs theorized as less or not related (divergent correlations) were negative, smaller, or nonsignificant. For example, none of the ChatGPT correlations between Warmth/Competence and the theoretically unrelated dimensions of Body (i.e. various terms relating to body parts and properties; e.g. “eyes,” “legs”) and nonfluencies (i.e. nonwords used in verbal communications; e.g. “uh,” “er”) were statistically significant. Results for SBERT were less robust, including smaller differences between convergent and divergent correlations. This pattern may suggest that stereotypes in SBERT (vs. ChatGPT), as retrieved via top words, align relatively less with human representations of the same social categories.

**Table 3.** Convergent (same-dimension) and divergent (cross-dimension) correlations between language model and human responses for ChatGPT and SBERT.

Property	Dimension	Human stereotypes					
		Direction		Representativeness		Other	
		Warmth	Competence	Warmth	Competence	Body	Nonfluency
ChatGPT							
Direction	Warmth	0.352**	0.160**	−0.045	0.074	−0.014	0.019
	Competence	0.185**	0.501**	0.073	0.173**	−0.029	−0.050
Representativeness	Warmth	0.041	−0.179**	0.442**	−0.264**	−0.077	−0.039
	Competence	0.043	0.195**	−0.259**	0.503**	0.072	−0.062
SBERT							
Direction	Warmth	0.118**	0.096*	−0.039	0.075*	0.003	−0.018
	Competence	0.101*	0.184**	−0.037	0.130**	0.060	−0.090*
Representativeness	Warmth	−0.282**	−0.236**	0.390**	−0.227**	−0.042	−0.044
	Competence	0.029	0.008	−0.113*	0.217**	−0.075*	−0.030

Bolded values indicate the correlations relevant to convergent validity.

Correlations conducted on human dictionary prevalence and direction scores.

*P < 0.05.

**P < 0.001.

These results initially establish that language models are capturing not only stereotypes' directionality, but also their representativeness, in ways similar to human stereotypes, and that we can measure these using the same instruments as in human data (e.g. dictionaries).

### Improvement in human bias detection

Finally, we examine whether we can improve the correspondence between human and language model category representations by using both direction and representativeness variables. Thus, we further validate the distinction between direction and representativeness biases in language models by testing if they are independent predictors of human general valence evaluations.

First, a model adding representativeness measures outperformed a model including only direction (see Table 4). The patterns suggest that higher Warmth representativeness predicts more negative human evaluations of the category, while higher Competence representativeness predicts more positive human evaluations (in ChatGPT only, *n.s.* for SBERT), when controlling for the direction of the dimension, again in line with the negativity of Warmth and positivity of Competence stereotypes.

**Table 4.** Nested models showing incremental validity of Warmth and competence representativeness and its interaction with direction, for ChatGPT and SBERT, in predictions of human ratings of valence.

Model	#	Predictor	*b*	*t*	*P*	*R* ^2^	AIC	*χ* ^2^	*P*
ChatGPT	**1**	**Warmth direction**	0.262	5.98	<0.001				
		**Competence direction**	0.345	5.18	<0.001	0.171	667.10		
	**2**	Warmth direction	0.263	6.05	<0.001				
		Competence direction	0.303	4.59	<0.001				
		**Warmth representativeness**	−0.415	−2.72	0.007				
		**Competence representativeness**	0.398	3.02	0.003	0.227	647.29	23.81	<0.001
	**3**	Warmth direction	−0.082	−1.29	0.197				
		Competence direction	0.242	2.43	0.016				
		Warmth representativeness	−0.421	−2.90	0.004				
		Competence representativeness	0.186	0.33	0.333				
		**Warmth interaction**	1.198	7.18	<0.001				
		**Competence interaction**	0.280	1.22	0.222	0.322	600.74	50.55	<0.001
SBERT	**1**	**Warmth direction**	0.167	4.47	<0.001				
		**Competence direction**	0.058	1.45	0.148	0.027	848.43		
	**2**	Warmth direction	0.106	2.87	0.004				
		Competence direction	0.043	1.10	0.271				
		**Warmth representativeness**	−1.445	−7.08	<0.001				
		**Competence representativeness**	0.075	0.28	0.781	0.13	804.12	48.31	<0.001
	**3**	Warmth direction	0.006	0.14	0.892				
		Competence direction	−0.086	−1.75	0.081				
		Warmth representativeness	−0.766	−2.88	0.004				
		Competence representativeness	−0.756	−2.21	0.028				
		**Warmth interaction**	1.007	3.12	0.002				
		**Competence interaction**	1.684	3.73	<0.001	0.167	783.34	24.78	<0.001

Model comparison metrics are provided, comparing the three levels of regression for each language model. Higher values for the outcome indicate more positive human ratings of the social categories.

**Fig. 4.**
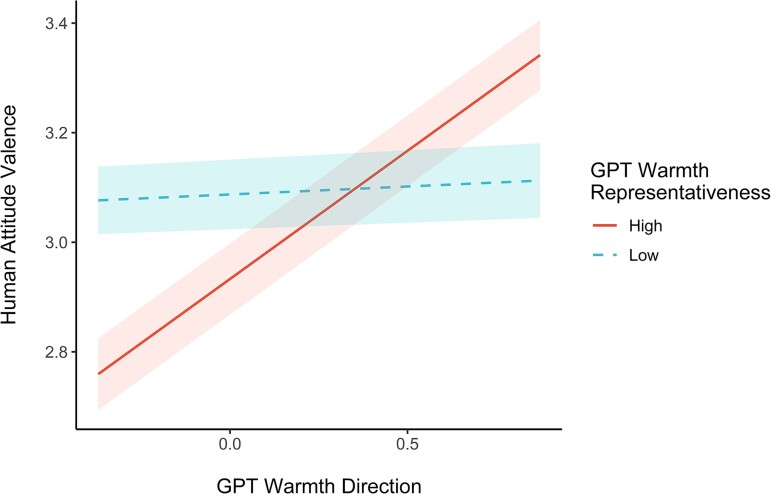
Interaction between ChatGPT Warmth representativeness and direction in predicting human valence evaluations. Representativeness shown at ±1 SD from the mean. The error ribbons represent ±1 SEs. Higher Human Attitude Valence indicates more positive global evaluations in human data.

This pattern is further extended to interactive effects between representativeness and direction. These interactive models improved upon their additive versions. For example, the interaction for ChatGPT suggests that directional biases for Warmth are better predictors of general human bias when Warmth is more representative of the category (see Fig. 4; a similar pattern occurs in SBERT). Thus, capturing human biases in language models is better achieved when both direction and representativeness are accounted for.

## Discussion

The current study takes an interdisciplinary approach to integrate recent developments in stereotyping theories with research on bias in AI. Specifically, we introduce the construct of stereotype representativeness as a measure of association with a content dimension for the identification of bias in two recent language models: GPT 3.5 Turbo as implemented in ChatGPT and SBERT. We provide evidence for the construct differentiability of representativeness and existing measures of directionality and illustrate consequences of these two axes of bias. We focus on the “big two” dimensions of Warmth and Competence to examine these patterns.

We first provided evidence that direction left substantial variance unexplained in a measure of representativeness, in support of representativeness–direction differentiability. To illustrate and further establish these patterns, we then looked at specific social categories. Using both an extensive list of 87 social categories^c^ and a list of racialized surnames for White, Asian, and Hispanic targets, we show how various targets exhibit differentiability in their direction and representativeness scores. For example, Hispanic and White surnames had similarly high direction scores for Competence, but Competence was more representative of White than Hispanic surnames. Comparably, associations for “welfare recipients” and “unemployed” individuals had similarly low Warmth direction scores, but differed in representativeness, such that the model associated “welfare recipients” with Warmth more often. These findings illustrate how ignorance of representativeness excludes a significant axis of bias in language models' representations of social categories.

Next, we tested the extent to which Warmth and Competence representativeness and direction correlate with the models' internal representations of general valence. General valence is an important metric, similar to attitude measures, that provides broad information about how positively or negatively a category is represented. Indeed, Warmth and Competence direction measures positively correlated with general valence. This pattern aligns with human data, where Warmth and Competence are central evaluative components (28), and where direction and valence are often highly correlated (1). More relevantly, as expected, adding Warmth and Competence representativeness, both as independent covariates and as interactive terms with direction, improved predictions of internal general valence. In other words, both representativeness and direction provide relevant information about the overall AI models' valence representation of the categories.

In order to examine whether the language models represented social categories similarly to humans across both constructs, we correlated the ChatGPT and SBERT data with human stereotypes. We found that, as expected, representativeness and direction scores in the language models positively correlated with their corresponding scores in human data and showed smaller correlations with less-related constructs (e.g. stereotypes about body properties). This pattern provided preliminary evidence that these AI measures were behaving similarly to human data, where previous research had demonstrated direction-representativeness differentiability (1). In addition, these patterns align with convergent and divergent validity expectations for our measures.

Finally, we examined whether we can further improve congruence with human data by using information about both representativeness and direction from language models' representations. We again find that beyond direction, representativeness explained significant variance in Americans' prejudice toward social categories. For example, Warmth direction was a better predictor of human general valence evaluations of a social category when Warmth was a representative stereotype of the category, paralleling the relationship between representativeness, direction, and general prejudice in human data (1).

Across all analyses, thus, we find substantial and robust evidence for the differentiability of representativeness and direction as properties of stereotypical biases in language models. This finding has both theoretical and practical implications. At the theoretical level, our results suggest that higher correspondence between measures of AI bias and human stereotypes can be achieved by including measures of both direction and representativeness. An increasing number of studies seek to gain insights into human biases by exploring AI models as reflections of human culture. For example, recent studies using language models have contributed to theories about stereotype change over time (17), androcentrism (29), and dehumanization (30). While challenges remain for improving inferences from AI patterns to social psychological theories (31), our study suggests that incorporating multiple stereotype properties into similar studies, beyond the commonly used measures of direction, may further increase congruence with measures of social perceptions and improve the impact of AI-based results on our understanding of human bias.

At the practical level, higher congruence between human and AI stereotype measures may improve bias auditing by allowing better comparisons to human data as a benchmark for expected biases in AI models. Additionally, our results uncover representativeness as a property that may allow biases to persist beyond current auditing and debiasing approaches. Recent debiasing methods attempt to correct stereotypes by addressing direction imbalances (e.g. 24). While these methods are effective at reducing such directional biases, they do not aim as implemented to reduce the representativeness biases shown here. Certainly, these methods may indirectly reduce representativeness biases, but our findings suggest this should not be assumed, and debiasing methods should be explicitly tested on this variable. Additionally, other debiasing methods operate differently (e.g. by removing social category signals (32, 33)), with unclear implications for representativeness biases. We expect future research will adapt bias management methods to address representativeness more directly, as needed.

Our focus on Warmth and Competence highlights another aspect of stereotypes that has often been neglected: the dimensionality of content. Warmth and Competence have well-established centrality and prevalence in stereotypes, influencing outcomes from emotional responses to decision-making regarding social categories (e.g. 28, 34). Importantly, many social categories are stereotyped as high on one dimension but low on the other, creating more complex patterns of stereotype that are missed with unidimensional measures of general valence. However, these dimensions have only recently started to receive attention in studies of fairness in AI (e.g. 23). Our results support that Warmth and Competence independently structured stereotypes in the language models examined, revealing biases that would not have emerged from more general measures of valence. This suggests that auditing and future studies of AI bias should consider Warmth and Competence content for a systematic evaluation of human stereotypes, along with other stereotype content dimensions (31).

In addition, we used two different language models and methods and find robust evidence for the direction–representativeness distinction. However, the specific biases and patterns that arose across models differed. For example, when looking at racialized surnames, ChatGPT showed higher Warmth representativeness for “Hispanic” than “Asian,” while the SBERT top words analysis had higher scores for “Asian” than “Hispanic.” Some of these patterns are likely a result of differences in training data for the models (e.g. it is possible that the surnames in each training data differ in their associations with Warmth). Other results may be a function of the different methods applied to each model (e.g. ChatGPT may not retrieve associations in the same way as we retrieved top words from SBERT; see Supplement for additional alternative analyses). Future research may further clarify these differences, but our main goal in including both models was to test the robustness of our differentiability hypothesis, which was supported by our results.

Finally, our study incorporated two approaches to target selection: a general approach using multiple social category terms, and a specific approach using exemplars (i.e. racialized surnames). The first has only recently been used in AI bias research (e.g. 17), despite a long tradition in social psychology due to its ability to explore patterns across categories (3). By examining both stimuli simultaneously, we were able to determine whether some patterns may differ based on category term vs. exemplar selection. Indeed, we found that biases that arose in associations with category terms did not necessarily arise for exemplars. Social category terms associations (vs. exemplars) may be more likely to include some definitional associations (e.g. status-related words associated with terms such as “rich” and “poor”; c.f., 35), which are not as relevant to bias. On the other hand, category exemplars such as names and surnames should not be essentialized as properties of a category (36) and may be more confounded by other associations. This suggests that auditing and debiasing methods should examine multiple types of targets (i.e. category terms and name exemplars, as well as other category signals, such as pronouns).

### Limitations and future directions

For congruence with basic cognitive models of stereotyping, the current studies focused on simple semantic associations. However, stereotypical associations may also occur through more complex patterns expressed in longer-text formats. We also focused on Warmth and Competence as the “big two” dimensions of stereotype content, but many other dimensions of stereotypes have been identified as prevalent in human stereotypes (e.g. Deviance, Health (1)). In the Supplement, we provide initial evidence for the separability of direction and representativeness in the political–religious Beliefs dimension (37, 38), but future research should test for the role of direction and representativeness along additional contents. In addition, we rely on English-language tests, requiring that future research replicates these patterns in other languages and cross-culturally relevant training sets. Another issue for future research is the role of frequency of occurrence of category and content dimension signals. Previous research (20) suggests that lower-frequency racialized names show more bias and overfitting. It is possible that representativeness relates to stimuli frequency patterns in ways that shed light on its relationship with general valence. Finally, future research should test additional AI models and targets (e.g. category intersections (39)) for further generalizability.

### Conclusion

In the current paper, we show that, across two widely used language models, direction and representativeness are differentiable components of stereotypical associations. This differentiability arises for both a large sample of social category terms and racialized surnames, and including both representativeness and direction metrics improves congruence with internal metrics of general valence and external metrics of human stereotypes. These findings suggest that current auditing methods fail to account for representativeness biases in stereotypes and provides insights for improving AI-to-human theoretical inferences and approaches to fairness and accountability in AI.

## Materials and methods

### Stimuli

We used two sets of social category signals: a list of surnames culturally associated with three racial categories (Asian, Hispanic, and White), and a large list of salient social category terms used in human stereotyping research (1). The first was retrieved from previous research on AI bias (40, 41). For the latter, the researcher team and three research assistants independently reviewed a preliminary list of 87 category terms (from 1) and compiled additional synonyms closely related to the categories. This resulted in a list of 1,366 different terms (including singular and plural forms) for 87 salient social categories in the United States. See the Supplement for a full list of categories and their terms (but note that derogatory terms are included for scientific reproducibility), as well as additional information and validation of stimuli selection.

For human data, we used a subset of the category terms, specifically removing singular forms and slurs. We had a total of 484 terms evaluated by participants, including at least one term for each of the 87 social categories.

### Language models, human data, and analytic procedure

#### ChatGPT

One of the examined models is GPT 3.5 turbo as implemented in freely available versions of ChatGPT as of 2024 June 12 (42). The ChatGPT model was trained on vast amounts of data, including the Common Crawl (a large scraping of internet webpages), books, Reddit, and Wikipedia (43), as well as human feedback in reinforcement learning (44), and potentially others.

We accessed the model through the Python OpenAI API. We used prompts that closely resembled those used with human participants (1). As a “system prompt,” we indicated: “You will be given information about a series of target groups of people. Answer how most people in American society would respond” to underscore our interest in cultural associations.

Then, to obtain the model's internal general valence representations of the categories, we also modeled the prompt after human data (1). Specifically, we prompted: “In general, how does American society view people who are [*target*]? Use a scale ranging from (1) Very negatively to (5) Very positively. Do not provide an explanation, only a single-number response using the scale.”

To obtain stereotypical associations for each target, we prompted: “List 50 characteristics that you believe most Americans would think describe [*target*]. Use single words.” The prompts are designed to align with human instructions in previous studies, where requesting cultural (vs. personal) stereotypes reduces social desirability (ChatGPT has moderation features resulting in more warnings for more direct prompts) while retaining high predictivity of bias and discriminatory intent (1, 34, 45). Our conclusions are robust to checks using a more direct system and content prompt (“List 50 characteristics that you believe describe [*target*];” see online repository).

To obtain the most deterministic results, we set the temperature to 0, and if repeated responses occurred per target, they were removed. The output sometimes included warnings about bias in the responses, which were removed. In addition to warnings, the API failed to return responses for category terms it indicated are “not commonly used or understood in American society” (e.g. “mahanaya”). For all 87 categories, except the “Black” category (which returned only warnings), the API provided the requested output for at least one term. This resulted in the removal of the “Black” category from ChatGPT analyses.

Having obtained the ChatGPT responses, we preprocessed them by transforming from plural to singular, removing capitalization, and replacing dashes with spaces. Then, we took a dictionary approach that has been used in human studies (e.g. 46). These dictionaries are lists of words coding for Warmth and Competence content that can be matched to the ChatGPT responses. We accessed the dictionaries via the R SADCAT package v1.1. The dictionaries have been validated (47) and shown to be predictive of relevant outcomes, such as decision-making and general prejudice, when coding human open-ended stereotypes (1).

The dictionaries code separately for representativeness and direction. For representativeness, a ChatGPT response that is present in a dictionary receives a score of 1 for the corresponding dimension (0 if absent). For example, if “sociable” or “unfriendly” are ChatGPT responses, they would receive a score of 1 for Warmth and 0 for Competence, since they are words in the Warmth but not Competence dictionaries. On the other hand, “smart” or “unintelligent” would be coded as Competence but not Warmth words for representativeness. Some words are present in both dictionaries and would be coded accordingly.

Having coded for representativeness, responses were scored for direction per dimension. Direction ranged from −1 (low) to 1 (high) but was coded as missing data if the response was not about the dimension (i.e. if it received a score of 0 for representativeness). Thus, responses such as “sociable” or “moral” received direction scores of 1 for Warmth, while “unfriendly” received a score of −1, and all were coded as missing values for Competence; “smart” has a score of 1 for Competence direction, while “unintelligent” has a score of −1, with missing values for Warmth. Because ChatGPT was prompted to provide 50 responses per term, for representativeness and direction scores we averaged across responses.

#### SBERT

We use SBERT (48) as an alternative model. The selected SBERT (“mpnet-base-v2”) was trained on multiple predominantly English sources (e.g. Reddit, Wikipedia, WikiAnswers; see https://huggingface.co/sentence-transformers/all-mpnet-base-v2). Examining SBERT provides additional generalizability evidence for our findings, as well as robustness to differences between the models. For example, SBERT is a base model, while ChatGPT has been fine-tuned as a chatbot. Thus, ChatGPT has additional training and debiasing methods applied to the output (49), as well as potential issues such as hallucinations (50).

Text embeddings underlie modern language models (51, 52), which, for SBERT, are open source (here, accessed through Python's SentenceTransformers module). Thus, here we use these embeddings, in line with previous research (17), to obtain top associations with the social categories and racialized surnames.

First, we retrieved an additional large list (*N* = 27,075) of general words (as in 17). Second, for each target (e.g. “nurse”) and word in the list (e.g. “warm,” “tall”), we obtain their SBERT embedding representation (768-dimensional numerical vectors). Then, we obtained the correlation (cosine similarities) between each target embedding and each general word embedding and sorted these results based on the correlations. We removed associations with correlations above 0.7 since these were largely synonyms of the stimuli (e.g. for “teenagers” such associations include “teenager,” “teens,” and “teenaged”). Then, we obtained the top 50 words most associated with each category. This way, we were able to use the same procedure used for ChatGPT for dictionary coding of representativeness and direction, in this case on the top words from SBERT embeddings correlations.

Computation of general valence metrics involved a different process, since we are interested in the overall internal representation of the targets by the model. Specifically, we used the text embeddings directly, in line with techniques such as the WEAT (14). First, we retrieved validated sets of positive and negative words (from 44) and obtained their SBERT embeddings. Subsequently, the embeddings for each set were averaged, resulting in embeddings for positive and negative words. Then, we subtracted the negativity embedding from the positivity embedding, resulting in a general valence embedding. For this general valence embedding, higher scores indicate more positivity and lower scores indicate more negativity. Finally, we obtained cosine similarities between each term embedding and the general valence embedding, which provides general valence scores for each social category term.

#### Human data

For comparisons with human patterns (e.g. testing predictions of human valence), we collected responses from a representative sample (in terms of race, gender, and age) of paid American residents via the online Prolific platform. Participants (*N* = 1,790) were first asked to “Please list four characteristics that you spontaneously think about the following type of person. Please use single words if possible, and not more than two per box. People who are [term].” These targets were the category terms described above, and each participant saw a random sample of three targets. We then analyzed these responses with the same approach as for the language models, using dictionary coding to extract representativeness and direction scores. These dictionary codes include scores for “Body/Body parts and properties” and “nonfluencies” (53). Higher scores for these variables indicate that the category had more responses coded as being about body parts, or nonfluencies (e.g. “uh,” “hm,” and “um”). These dimensions are relevant to understand stereotypes (e.g. Appearance is a relevant dimension of content, 1; nonfluencies may indicate lack of stereotype knowledge) but are not expected to correlate strongly with Warmth and Competence (see Supplement for additional information).

Subsequently, participants were asked: “in general, how does society view people who are [term]?” followed by a 1—Very negatively to 5—Very positively rating scale. This is the general valence evaluation metric. Participants were also asked for exploratory numerical ratings of various content dimensions' direction (e.g. political–religious Beliefs), which are not discussed here. Finally, participants provided demographics (mean age = 44.28; 48.4% men, 49.7% women, 1.6% nonbinary; 62% White, 13.4% Black, 6.4% Asian, 5.8% Hispanic, 9% multiracial).

For the main analyses, we averaged across participants, and used the average scores for each category term (each category term was evaluated by an average of 11.1 participants, SD = 3.68). We then matched human responses to the language model data by their corresponding terms, where available, resulting in 506 matched observations.

The study protocol was approved by the Rutgers University Institutional Review Board (Pro2023000238), and participants received informed consent prior to the study.

#### Regression models

In general, we use mixed regression models with category as a random factor and each term/exemplar as an observation. In analyses examining variance explained, we report the mixed models' marginal *R*^2^s. Large variance left unexplained would suggest that direction and representativeness are distinct constructs. To address the question that representativeness may relate to extremity on a content dimension, rather than direction alone, we also include direction squared in these models (i.e. a quadratic model).

When illustrating patterns with specific surnames or social categories, we use estimated marginal means and pairwise comparisons derived from mixed models predicting direction and representativeness from the stimuli. Our goal with these analyses is to illustrate that in the language models, a smaller difference in direction between specific categories may be accompanied by a larger difference in representativeness, highlighting the importance of examining both as independent stereotype properties. For this purpose, we present standardized effect sizes. We also report statistical significance, which aligns with these conclusions. But we caution interpretation of statistical significance in these specific analyses, given issues with interpretation of nonsignificant results and comparison against significant patterns (e.g. 54).

When comparing nested regressions (e.g. Table 3), we were interested in both the individual coefficients and the general model comparisons. For each ChatGPT and SBERT, we compare three nested regression models: a first level including only Warmth and Competence direction as predictors, a second level adding representativeness, and a third adding the direction-by-representativeness interaction terms. We present χ^2^ with statistical significance comparing each nested model and AIC values (to examine model improvement penalized for number of predictors), with a threshold of an AIC decrease of 2 points for sufficient model improvement (55).

Given the large number of observations, we had power >90% for most tests of interest, using a small-to-medium effect size (*r* = 0.2), as indicated by the R power simulation package *simr* (56). However, power for tests comparing pairs of specific social categories varied based on number of category terms.

## Supplementary Material

pgae493_Supplementary_Data

## Data Availability

All data, code, and materials are available at: https://osf.io/tvdxu/?view_only=1cfdbfcecee74ef494d3c55800253b11.

## Notes

a See previous research (e.g., 4) for discussion on when stereotypes may be, on average, “accurate.” Although we examine patterns across stereotypes, regardless of “accuracy,” we note that any stereotype may result in essentialized perceptions of social categories and other issues listed in the Introduction.

b However, the two constructs do not always correlate. Most Warmth and Competence research has focused on direction rather than valence, as we do, see (1).

c 86 for ChatGPT analyses, due to the model providing only warnings for the “Black” category terms.
