# Supplementary material for: Directionality and representativeness are differentiable components of stereotypes in large language models
Source: PNAS Nexus. 2024 Nov 4;3(11):pgae493. doi: 10.1093/pnasnexus/pgae493 (PMC11586767; doi:10.1093/pnasnexus/pgae493)
Supplement: pgae493_Supplementary_Data [file pgae493_supplementary_data.docx]

**Directionality and representativeness are differentiable components of**

**stereotypes in large language models**

Contents

[Additional Results 2](#_Toc180776598)

[Variance Explained – Coefficients 2](#_Toc180776599)

[Across-category separability – Full Figures 2](#_Toc180776600)

[Specific case illustration – Permutation tests 6](#_Toc180776601)

[Additional Figures for Interaction Effects (Tables 3 and 4, Main Text) 6](#_Toc180776602)

[Text Embedding Analysis of SBERT 14](#_Toc180776603)

[Exploration of effects for Beliefs as a third stereotype content 18](#_Toc180776604)

[Additional Methods 19](#_Toc180776605)

[Social Category Terms 19](#_Toc180776606)

[Racialized Surnames 36](#_Toc180776607)

[Additional Dictionary Information 37](#_Toc180776608)

[Additional Preprocessing Information 39](#_Toc180776609)

**Please note that the Supplemental Information and data repository contain offensive language (in the form of category terms used for data collection to capture broad, real-world representations of social categories), that neither NAS nor the authors condone.**

# Additional Results

## Variance Explained – Coefficients

Table S1. Additional model information – Variance Explained analyses (Representativeness outcome).

| Stimuli | Model | Dimension | Direction |  | Extremity |  |
| --- | --- | --- | --- | --- | --- | --- |
|  |  |  | *b* | *p* | *b* | *p* |
| Category terms | ChatGPT | Warmth | 0.006 | 0.777 | 0.117 | < .001 |
|  |  | Competence | 0.049 | 0.039 | 0.136 | < .001 |
|  | SBERT | Warmth | -0.036 | 0.001 | -0.021 | 0.123 |
|  |  | Competence | 0.031 | 0.006 | -0.05 | < .001 |
| Surnames | ChatGPT | Warmth | 0.193 | 0.083 | 0.071 | 0.567 |
|  |  | Competence | 4.332 | < .001 | -2.775 | < .001 |
|  | SBERT | Warmth | -0.027 | 0.663 | -0.073 | 0.314 |
|  |  | Competence | 0.016 | 0.046 | -0.026 | 0.045 |

##

## Across-category separability – Full Figures

Figure S1. Warmth Direction-Representativeness separability using a larger sample of social categories, ChatGPT





Figure S2. Competence Direction-Representativeness separability using a larger sample of social categories, ChatGPT





Figure S3. Warmth Direction-Representativeness separability using a larger sample of social categories, SBERT





Figure S4. Competence Direction-Representativeness separability using a larger sample of social categories, SBERT





## Specific case illustration – Permutation tests

Findings are replicated when using permutation tests instead of linear regressions (c.f., Caliskan et al., 2017) [see online repository independence tests].

##

## Additional Figures for Interaction Effects (Tables 3 and 4, Main Text)

Figure S5. Interaction between Warmth representativeness and direction in predictions of general internal valence, GPT





Figure S6. Interaction between Competence representativeness and direction in predictions of general internal valence, GPT





Figure S7. Interaction between Competence representativeness and direction in predictions of general human valence, GPT





Figure S8. Interaction between Warmth representativeness and direction in predictions of general internal valence, SBERT





Figure S9. Interaction between Competence representativeness and direction in predictions of general internal valence, SBERT





Figure S10. Interaction between Warmth representativeness and direction in predictions of general human valence, SBERT





Figure S11. Interaction between Competence representativeness and direction in predictions of general human valence, SBERT





## Text Embedding Analysis of SBERT

In an alternative analysis, we rely directly on the text embeddings, rather than first retrieving the top words. The computation of Warmth and Competence direction was similar to the main text computation of general valence: we obtained embeddings for prototypical sets of low and high direction words for each dimension (retrieved from 43), averaged each set, and subtracted them such that we obtained “direction embeddings” varying from low to high direction per dimension. These direction embeddings were then correlated (cosine similarity) with the target embeddings. These procedures follow a similar logic to existing metrics (e.g., WEAT, where the relative similarity of the target embedding to one direction vs. the other is obtained, 16), and has been previously used and validated (1).

The novel representativeness metric was obtained using a slightly different process: instead of a difference score, the target embeddings were correlated with an embedding averaging all the prototypical words for a dimension (balanced such that an equal number of high and low words were included). As such, higher cosine similarities indicate that the target is more associated with the dimension (rather than with one pole vs. the other). By computing similarity to a vector capturing the concepts of Warmth or Competence, this measure approximately indicates strength of association with the dimension, regardless of direction (see results for validity data). This method has also been used before and correlates with other measures of human content representativeness (1).

The comparison between dictionary coding and embeddings involves strengths and limitations. Dictionaries allow for a cleaner separation of representativeness and direction, while these properties are more correlated for embeddings (see results and 1). Working with ChatGPT top words is also more transparent and directly interpretable than working with embeddings. Additionally, we are aware of limitations regarding embedding extraction, anisotropy, and cosine similarity-based measurements (e.g., 47). However, we do not currently have a better approach to work with embeddings directly, and we will address these limitations as we improve embeddings-based approaches.

Nonetheless, the embeddings approach has advantages, compared to ChatGPT elicitations and dictionary coding. For example, embeddings are arguably more comprehensive, as they capture all of the model’s semantic information in relation to the target. Additionally, embeddings provide continuous (vs. all-or-nothing) metrics of semantic similarity that better approach language use, and may facilitate scaling, automation, and generalization (as embeddings underlie contemporary language models). The contextualized embeddings we use here also allow for domain-relevant sense disambiguation (e.g., “warmth” should refer to a psychological trait not a physical trait). However, given the contextualization in the ChatGPT elicitation task and the fact that the dictionaries were developed for the stereotyping domain (43), this is less of an issue here. An additional difference is that ChatGPT has additional debiasing methods applied to the output (48), while the SBERT model used here does not. Finally, the ChatGPT approach may have additional limitations, such as hallucinations (49), which can also be further addressed in future research.

Some results using this approach include that, in line with our separability hypothesis, a quadratic model for ChatGPT’s Warmth direction accounted for a minority of the variance in Warmth representativeness (*R^2^* = .068). A similar model for Competence accounted for .087 of the representativeness variance. In addition, we present some specific examples of separability, and results for prediction of general valence, which showed that the more complex models improved predictions (although the interaction patterns were sometimes inversed, potentially due to high correlations between representativeness and direction indicators).

Table S2. Pairwise surname comparisons per dimension for direction and representativeness.

| Comparison | Dimension | *P*s |
| --- | --- | --- |
| Black vs. Asian | Warmth | Direction = .092  Representativeness = < .001 |
| Working-class vs. middle-class | Warmth | Direction = .732  Representativeness = .046 |
| Black vs. Hispanic | Competence | Direction = .661  Representativeness = < .001 |
| Scientists vs. Athletes | Competence | Direction = .272  Representativeness = < .001 |
| Hispanic vs. Native American | Warmth | Direction = .135  Representativeness = < .001 |
| Asian vs. Middle Eastern | Competence | Direction = .091  Representativeness = .038 |

Table S3. Internal Valence prediction using SBERT embeddings method

| **Model** | **#** | **Predictor** | ***b*** | ***t*** | ***p*** |  | ***R2*** | ***AIC*** | ***χ2*** | ***p*** |
| --- | --- | --- | --- | --- | --- | --- | --- | --- | --- | --- |
| SBERT | **1** | **Warmth Direction** | 0.36 | 19.17 | < .001 |  |  |  |  |  |
|  |  | **Competence Direction** | 0.46 | 26.01 | < .001 |  | 0.669 | -5113.1 |  |  |
|  | **2** | Warmth Direction | 0.4 | 21.43 | < .001 |  |  |  |  |  |
|  |  | Competence Direction | 0.395 | 21.52 | < .001 |  |  |  |  |  |
|  |  | **Warmth Representativeness** | -0.26 | -10.09 | < .001 |  |  |  |  |  |
|  |  | **Competence Representativeness** | 0.233 | 9.14 | < .001 |  | 0.697 | -5208.5 | 99.46 | < .001 |
|  | **3** | Warmth Direction | 0.223 | 3.87 | < .001 |  |  |  |  |  |
|  |  | Competence Direction | 0.585 | 10.92 | < .001 |  |  |  |  |  |
|  |  | Warmth Representativeness | -0.264 | -10.26 | < .001 |  |  |  |  |  |
|  |  | Competence Representativeness | 0.235 | 9.24 | < .001 |  |  |  |  |  |
|  |  | **Warmth Interaction** | 0.511 | 3.3 | .001 |  |  |  |  |  |
|  |  | **Competence Interaction** | -0.536 | -3.85 | < .001 |  | 0.699 | -5222.1 | 17.53 | < .001 |

Table S4. Human Valence prediction using SBERT embeddings method

| **Model** | **#** | **Predictor** | ***b*** | ***t*** | ***p*** |  | ***R2*** | ***AIC*** | ***Sum of Sq*** | ***p*** |
| --- | --- | --- | --- | --- | --- | --- | --- | --- | --- | --- |
| SBERT | **1** | **Warmth Direction** | 8.35 | 5.52 | < .001 |  |  |  |  |  |
|  |  | **Competence Direction** | 5.91 | 5.5 | < .001 |  | 0.329 | 166.71 |  |  |
|  | **2** | Warmth Direction | 8.896 | 6.7 | < .001 |  |  |  |  |  |
|  |  | Competence Direction | 5.18 | 4.77 | < .001 |  |  |  |  |  |
|  |  | **Warmth Representativeness** | -4.57 | 2.27 | .026 |  |  |  |  |  |
|  |  | **Competence Representativeness** | 6.8 | 4.18 | < .001 |  | 0.42 | 156.99 | 4.61 | < .001 |
|  | **3** | Warmth Direction | 29.09 | 4.53 | < .001 |  |  |  |  |  |
|  |  | Competence Direction | 20.32 | 3.22 | .002 |  |  |  |  |  |
|  |  | Warmth Representativeness | -2.95 | 1.49 | .14 |  |  |  |  |  |
|  |  | Competence Representativeness | 5.95 | 3.64 | < .001 |  |  |  |  |  |
|  |  | **Warmth Interaction** | -55.09 | 3.2 | .002 |  |  |  |  |  |
|  |  | **Competence Interaction** | -39.96 | 2.59 | .011 |  | 0.474 | 151.22 | 2.87 | .009 |

## Exploration of effects for Beliefs as a third stereotype content

Although Warmth and Competence are the most prevalent and influential stereotypes, there are other dimensions that people use to stereotype social categories. Beliefs is one such dimension that has been explored very recently in LLMs. Here we show that the separability of representativeness and direction occurs for this dimension as well, as in human data, using ChatGPT.

First, Beliefs direction and extremity did not explain much variance in Beliefs representativeness, R^2^ = .043.

Second, we show the separability across groups in Figure S12

Figure S12. Beliefs Direction-Representativeness separability using a sample of social categories, ChatGPT





Third, although Beliefs direction did not predict ChatGPT internal valence (similarly to human data, since Beliefs ranges from conservative to progressive, showing no bias on this political-religious dimension). However, representativeness by itself did predict general valence, showing that higher Beliefs content, regardless of direction, correlates with lower valence.

# Additional Methods

## Social Category Terms

Table S5. List of social categories, their terms, and whether a warning was returned from the prompts.

| Category | term | Warning |
| --- | --- | --- |
| Accountants | bookkeepers | No |
| Accountants | actuaries | No |
| Accountants | analysts | No |
| Accountants | accountants | No |
| Accountants | auditors | No |
| Accountants | tellers | No |
| Adults | of age | No |
| Adults | grown up | No |
| Adults | grown-ups | No |
| Adults | grown ups | No |
| Adults | adults | No |
| Americans | American | No |
| Americans | North-American | No |
| Americans | US-americans | No |
| Americans | Usonians | No |
| Americans | yankees | No |
| Americans | gringos | No |
| Artists | artists | No |
| Artists | virtuosos | No |
| Artists | artisans | No |
| Asians | Filipino | No |
| Asians | Vietnamese | No |
| Asians | Asian | No |
| Asians | far easterns | No |
| Asians | far eastern | No |
| Asians | Taiwanese | No |
| Asians | Japanese | No |
| Asians | Korean | No |
| Asians | Chinese | No |
| Asians | East Asian | No |
| Asians | oriental | Yes |
| Asians | orientals | Yes |
| Atheists | agnostic | No |
| Atheists | non-believers | No |
| Atheists | pagans | No |
| Atheists | agnostics | No |
| Atheists | nonbelievers | No |
| Atheists | disbelievers | No |
| Atheists | atheists | No |
| Atheists | heretics | No |
| Atheists | atheist | No |
| Atheists | skeptics | No |
| Atheists | heathens | Yes |
| Atheists | infidels | Yes |
| Athletes | athletes | No |
| Athletes | athletic | No |
| Athletes | gymnasts | No |
| Athletes | sportspeople | No |
| Athletes | runners | No |
| Athletes | sportswomen | No |
| Athletes | sportsmen | No |
| Athletes | sprinters | No |
| Athletes | varsity men | No |
| Athletes | racers | No |
| Athletes | sports players | No |
| Bankers | brokers | No |
| Bankers | financiers | No |
| Bankers | bankers | No |
| Bisexuals | bisexual | No |
| Bisexuals | bi | No |
| Blind | sightless | No |
| Blind | legally blind | No |
| Blind | partially sighted | No |
| Blind | visually impaired | No |
| Blind | unsighted | No |
| Blind | blind | No |
| Blind | unseeing | No |
| Blue-collar | waged | No |
| Blue-collar | lower class | No |
| Blue-collar | lunchpails | No |
| Blue-collar | proletarian | No |
| Blue-collar | proletarians | No |
| Blue-collar | manual workers | No |
| Blue-collar | blue-collar | No |
| Buddhists | buddhists | No |
| Buddhists | buddhist | No |
| Buddhists | zens | No |
| Buddhists | theravadas | No |
| Buddhists | mahayanas | Yes |
| Buddhists | hinayanas | Yes |
| Buddhists | hinayana | Yes |
| CEOs | big shots | No |
| CEOs | bosses | No |
| CEOs | directors | No |
| CEOs | co-founders | No |
| CEOs | ceos | No |
| CEOs | executive directors | No |
| CEOs | entrepreneurs | No |
| CEOs | presidents | No |
| CEOs | capitalists | No |
| CEOs | barons | No |
| CEOs | tycoons | No |
| CEOs | bureaucrats | No |
| Catholics | churchgoing | No |
| Catholics | catholic | No |
| Catholics | roman catholics | No |
| Catholics | roman catholic | No |
| Catholics | catholics | No |
| Catholics | papists | Yes |
| Celebrities | idols | No |
| Celebrities | superstars | No |
| Celebrities | vips | No |
| Celebrities | luminaries | No |
| Celebrities | stars | No |
| Celebrities | famous | No |
| Celebrities | moguls | No |
| Celebrities | personalities | No |
| Celebrities | influencers | No |
| Celebrities | a-listers | No |
| Celebrities | celebrities | No |
| Celebrities | household names | No |
| Children | schoolboys | No |
| Children | toddlers | No |
| Children | kids | No |
| Children | juniors | No |
| Children | schoolgirls | No |
| Children | children | No |
| Children | rugrats | No |
| Children | elementary schoolers | No |
| Children | preschoolers | No |
| Children | infants | No |
| Children | juveniles | No |
| Children | anklebiters | No |
| Christians | christians | No |
| Christians | baptismals | No |
| Christians | christian | No |
| Christians | protestants | No |
| Christians | evangelicals | No |
| Christians | evangelical | No |
| Christians | baptismal | No |
| Conservatives | old-fashioned | No |
| Conservatives | conservatives | No |
| Conservatives | fundamentalists | No |
| Conservatives | conservative | No |
| Conservatives | establishmentarian | No |
| Conservatives | unprogressive | No |
| Conservatives | establishmentarians | No |
| Criminals | convicts | No |
| Criminals | offendors | No |
| Criminals | villains | No |
| Criminals | offenders | No |
| Criminals | felons | No |
| Criminals | delinquents | No |
| Criminals | thugs | No |
| Criminals | criminals | No |
| Criminals | outlaws | No |
| Criminals | lawbreakers | No |
| Criminals | thieves | No |
| Criminals | crooks | No |
| Criminals | culprits | No |
| Crossdressers | female impersonators | No |
| Crossdressers | crossdressers | No |
| Crossdressers | gender benders | No |
| Crossdressers | drag kings | No |
| Crossdressers | drag queens | No |
| Crossdressers | transvestites | Yes |
| Democrats | social justice warriors | No |
| Democrats | progressives | No |
| Democrats | communists | No |
| Democrats | socialists | No |
| Democrats | leftists | No |
| Democrats | democrats | No |
| Democrats | left-wingers | No |
| Democrats | progressive | No |
| Democrats | snowflakes | No |
| Disabled | maimed | No |
| Disabled | disabled | No |
| Disabled | differently abled | No |
| Disabled | quadriplegic | No |
| Disabled | paraplegic | No |
| Disabled | paralyzed | No |
| Disabled | handicapped | No |
| Doctors | physicians | No |
| Doctors | MDs | No |
| Doctors | clinicians | No |
| Doctors | medical practitioners | No |
| Doctors | surgeons | No |
| Doctors | doctors | No |
| Doctors | pediatricians | No |
| Drug addicts | crackheads | No |
| Drug addicts | dopes | No |
| Drug addicts | drug addicts | No |
| Drug addicts | drug users | No |
| Drug addicts | drug addicted | No |
| Drug addicts | trippers | No |
| Drug addicts | snowbirds | No |
| Drug addicts | drug abusers | No |
| Drug addicts | druggies | No |
| Drug addicts | pill poppers | No |
| Drug addicts | dopers | No |
| Drug addicts | hopheads | No |
| Drug addicts | junkies | No |
| Drug addicts | dopeheads | No |
| Drug addicts | methheads | No |
| Drug addicts | stoners | No |
| Drug addicts | burnouts | No |
| Educated | learned | No |
| Educated | educated | No |
| Educated | erudite | No |
| Educated | graduates | No |
| Educated | erudites | No |
| Educated | literate | No |
| Elderly | seniors | No |
| Elderly | geriatric | No |
| Elderly | senile | No |
| Elderly | aging | No |
| Elderly | retirees | No |
| Elderly | aged | No |
| Elderly | geezers | No |
| Elderly | old | No |
| Elderly | elderly | No |
| Elderly | fogeys | No |
| Elderly | senior citizens | No |
| Elderly | gray | No |
| Elderly | codgers | No |
| Engineers | aerospace engineers | No |
| Engineers | civil engineers | No |
| Engineers | chemical enginners | No |
| Engineers | electrical engineers | No |
| Engineers | engineers | No |
| Engineers | developers | No |
| Engineers | planners | No |
| Engineers | builders | No |
| Engineers | software engineers | No |
| Engineers | mechanial engineers | No |
| Engineers | computer engineers | No |
| Engineers | biomedical engineers | No |
| Engineers | designers | No |
| Engineers | architects | No |
| Gamers | technophiles | No |
| Gamers | programmers | No |
| Gamers | gamers | No |
| Gamers | techies | No |
| Gamers | tech gurus | No |
| Gay | lgbtq | No |
| Gay | lgbt | No |
| Gay | homosexuals | No |
| Gay | homosexual | No |
| Gay | queer | No |
| Gay | gay | No |
| Gay | queers | Yes |
| Geeks | geeks | No |
| Geeks | brainiacs | No |
| Geeks | bookworms | No |
| Geeks | dorks | No |
| Germans | deutsches | No |
| Germans | deutsch | No |
| Germans | German | No |
| Germans | kraut | Yes |
| Germans | jerries | Yes |
| Germans | fritzs | Yes |
| Germans | krautheads | Yes |
| Germans | krauts | Yes |
| Germans | heinies | Yes |
| Germans | huns | Yes |
| Goths | emos | No |
| Goths | steampunks | No |
| Goths | punks | No |
| Goths | goth | No |
| Goths | emo | No |
| Goths | goths | No |
| Goths | grunge | No |
| Goths | punk-rock | No |
| Hackers | hacktivists | No |
| Hackers | it specialists | No |
| Hackers | hackers | No |
| Hackers | coders | No |
| Hackers | cybercriminals | No |
| Heterosexuals | het | No |
| Heterosexuals | heteros | No |
| Heterosexuals | hets | No |
| Heterosexuals | straight | No |
| Heterosexuals | hetero | No |
| Heterosexuals | heterosexual | No |
| Heterosexuals | heterosexuals | No |
| Heterosexuals | breeders | No |
| Hindus | hindu | No |
| Hindus | hindus | No |
| Hindus | hindis | No |
| Hindus | vaishnavas | Yes |
| Hindus | vaishnava | Yes |
| Hippies | beatniks | No |
| Hippies | tree huggers | No |
| Hippies | yippies | No |
| Hippies | tree-huggers | No |
| Hippies | bohemians | No |
| Hippies | free spirits | No |
| Hippies | bohemian | No |
| Hippies | bohos | No |
| Hippies | peaceniks | No |
| Hippies | flower children | No |
| Hippies | hippies | No |
| Hippies | flower-children | No |
| Hipsters | fashionistas | No |
| Hipsters | hipsters | No |
| Hispanics | Latina | No |
| Hispanics | latin-american | No |
| Hispanics | hispanic | No |
| Hispanics | Latino | No |
| Hispanics | latin-americans | No |
| Hispanics | Latin | No |
| Hispanics | spanish-speaking | No |
| Hispanics | latin american | No |
| Home-schooled | home-schooled | No |
| Home-schooled | schooled-at-home | No |
| Home-schooled | self-taught | No |
| Home-schooled | self-educated | No |
| Homeless | vagrants | No |
| Homeless | homeless | No |
| Homeless | panhandlers | No |
| Homeless | unsheltered | No |
| Homeless | nomads | No |
| Homeless | nomad | No |
| Homeless | unhoused | No |
| Homeless | drifters | No |
| Homeless | hobos | No |
| Immigrants | aliens | No |
| Immigrants | foreigners | No |
| Immigrants | foreign | No |
| Immigrants | settlers | No |
| Immigrants | naturalized citizens | No |
| Immigrants | migrants | No |
| Immigrants | emigrants | No |
| Immigrants | immigrants | No |
| Immigrants | nonnatives | No |
| Immigrants | nonnative | No |
| Independents | third party | No |
| Independents | independents | No |
| Independents | politically independent | No |
| Independents | politically unaffiliated | No |
| Indians | Indian-american | No |
| Indians | Indian | No |
| Indians | South Asian | No |
| Indians | mahanayas | Yes |
| Indians | mahanaya | Yes |
| Investors | angel funders | No |
| Investors | venture capitalists | No |
| Investors | shareholders | No |
| Investors | industrialists | No |
| Investors | angel investors | No |
| Investors | stockholders | No |
| Investors | lenders | No |
| Investors | investors | No |
| Investors | businesspeople | No |
| Ivy-leaguers | ivy-leaguers | No |
| Ivy-leaguers | highbrows | No |
| Ivy-leaguers | posh | No |
| Ivy-leaguers | elites | No |
| Ivy-leaguers | elite | No |
| Ivy-leaguers | highbrow | No |
| Jews | israelites | No |
| Jews | hebrew | No |
| Jews | israelite | No |
| Jews | isrealites | No |
| Jews | judaistic | No |
| Jews | sephardis | No |
| Jews | hasidic | No |
| Jews | hebrews | No |
| Jews | judaists | No |
| Jews | hasidics | No |
| Jews | semitic | Yes |
| Jews | semitics | Yes |
| Jews | semites | Yes |
| Jews | judaistics | Yes |
| Jews | Jewish | Yes |
| Jews | jews | Yes |
| Jocks | brawns | No |
| Jocks | gymrats | No |
| Jocks | jocks | No |
| Jocks | brawn | No |
| Jocks | chads | No |
| Jocks | meatheads | No |
| Lawyers | counsels | No |
| Lawyers | attorneys | No |
| Lawyers | legal practitioners | No |
| Lawyers | lawyers | No |
| Lawyers | judges | No |
| Lawyers | legal advisors | No |
| Lawyers | shysters | No |
| Lesbians | lesbians | No |
| Lesbians | wlws | No |
| Lesbians | sapphics | No |
| Lesbians | dykes | Yes |
| Liberals | sjws | No |
| Liberals | feminists | No |
| Liberals | liberals | No |
| Liberals | left leaning | No |
| Liberals | liberal | No |
| Libertarians | libertarian | No |
| Lower-class | needy | No |
| Lower-class | lower-class | No |
| Lower-class | in need | No |
| Lower-class | bankrupt | No |
| Lower-class | working class | No |
| Lower-class | proletariat | No |
| Lower-class | low socioeconomic status | No |
| Lower-class | plebians | No |
| Men | joes | No |
| Men | boys | No |
| Men | fellows | No |
| Men | husbands | No |
| Men | dudes | No |
| Men | guys | No |
| Men | chaps | No |
| Men | male | No |
| Men | men | No |
| Men | bros | No |
| Men | gentlemen | No |
| Men | blokes | Yes |
| Mentally Handicapped | autistic | No |
| Mentally Handicapped | intellectually delayed | No |
| Mentally Handicapped | developmentally delayed | No |
| Mentally Handicapped | retarded | Yes |
| Mentally Handicapped | mentally handicapped | Yes |
| Mexicans | chicanos | No |
| Mexicans | chicana | No |
| Mexicans | chicanas | No |
| Mexicans | chicano | No |
| Mexicans | cholos | No |
| Mexicans | texmex | No |
| Mexicans | Mexican | Yes |
| Mexicans | brazers | Yes |
| Mexicans | chiapas | Yes |
| Middle Eastern | Eurasian | No |
| Middle Eastern | middle easterners | No |
| Middle Eastern | middle eastern | No |
| Middle Eastern | mideastern | No |
| Middle Eastern | Pakistani | No |
| Middle Eastern | Arabs | Yes |
| Middle Eastern | arab | Yes |
| Middle-class | middle-class | No |
| Middle-class | middle-income | No |
| Middle-class | bourgeois | No |
| Middle-class | middle america | No |
| Musicians | instrumentalists | No |
| Musicians | composers | No |
| Musicians | musicians | No |
| Musicians | vocalists | No |
| Musicians | singers | No |
| Musicians | rockers | No |
| Musicians | soloists | No |
| Musicians | performers | No |
| Muslims | hadjis | No |
| Muslims | sunni | No |
| Muslims | shias | No |
| Muslims | shiites | No |
| Muslims | muslim | No |
| Muslims | hijabis | No |
| Muslims | hijabi | No |
| Muslims | sunnis | No |
| Muslims | Islamic | Yes |
| Muslims | hajis | Yes |
| Muslims | haji | Yes |
| Muslims | shia | Yes |
| Muslims | shiite | Yes |
| Muslims | qadiani | Yes |
| Native Americans | natives | No |
| Native Americans | aboriginal | No |
| Native Americans | Native American | No |
| Native Americans | American indians | No |
| Native Americans | american indian | No |
| Native Americans | native | No |
| Native Americans | navajo | No |
| Native Americans | first nation | No |
| Native Americans | navajos | No |
| Native Americans | redskins | Yes |
| Native Americans | squaw | Yes |
| Native Americans | aboriginals | Yes |
| Native Americans | injuns | Yes |
| Nerds | nerds | No |
| Nurses | candy-stripers | No |
| Nurses | medical attendant | No |
| Nurses | RNs | No |
| Nurses | nurses | No |
| Nurses | bsns | No |
| Obese | plumps | No |
| Obese | tubby | No |
| Obese | chubby | No |
| Obese | stout | No |
| Obese | plump | No |
| Obese | flabby | No |
| Obese | pudgy | No |
| Obese | rotund | No |
| Obese | portly | No |
| Obese | big | No |
| Obese | paunchy | No |
| Obese | overweight | No |
| Obese | pudgies | No |
| Obese | fat | No |
| Obese | obese | No |
| Obese | heavy | No |
| Obese | tubbies | No |
| Obese | fat | No |
| Obese | porkos | Yes |
| Obese | fatties | Yes |
| Obese | flabbies | Yes |
| Obese | tubs | Yes |
| Obese | paunchies | Yes |
| Parents | parent | No |
| Parents | stepparents | No |
| Parents | genitors | No |
| Parents | biological parents | No |
| Parents | mothers | No |
| Parents | surrogates | No |
| Parents | fathers | No |
| Parents | progenitors | No |
| Parents | parents | No |
| Parents | guardians | No |
| Parents | surrogate mothers | No |
| Politicians | congresswomen | No |
| Politicians | lawmakers | No |
| Politicians | legislators | No |
| Politicians | officeholders | No |
| Politicians | senators | No |
| Politicians | public servants | No |
| Politicians | statesmen | No |
| Politicians | stateswomen | No |
| Politicians | congressmen | No |
| Politicians | politicians | No |
| Poor | beggars | No |
| Poor | penniless | No |
| Poor | poor | No |
| Poor | impoverished | No |
| Poor | destitute | No |
| Poor | destitutes | No |
| Poor | needy | No |
| Poor | low ses | No |
| Poor | badly off | No |
| Poor | broke | No |
| Preps | preppy | No |
| Preps | yuppies | No |
| Preps | hoity-toities | No |
| Preps | stuck-up | No |
| Preps | snobbies | No |
| Preps | hoity-toity | No |
| Preps | classies | No |
| Preps | cliques | No |
| Preps | spoiled | No |
| Preps | snobby | No |
| Preps | preps | No |
| Preps | preppies | No |
| Prostitutes | prostitutes | No |
| Prostitutes | sex workers | No |
| Prostitutes | streetwalkers | No |
| Prostitutes | escorts | No |
| Prostitutes | gigolos | No |
| Prostitutes | floozies | No |
| Prostitutes | call girls | Yes |
| Prostitutes | hookers | Yes |
| Prostitutes | hos | Yes |
| Prostitutes | whores | Yes |
| Prostitutes | harlots | Yes |
| Prostitutes | ladies of the night | Yes |
| Prostitutes | call boys | Yes |
| Rednecks | hayseeds | No |
| Rednecks | provinicial | No |
| Rednecks | rednecks | No |
| Rednecks | bumpkins | No |
| Rednecks | provinicials | No |
| Rednecks | hillbillies | No |
| Rednecks | hicks | No |
| Religious | spiritual | No |
| Religious | churchgoers | No |
| Religious | practicing | No |
| Religious | religious | No |
| Religious | orthodox | No |
| Religious | religiously devout | No |
| Republicans | traditional | No |
| Republicans | republicans | No |
| Republicans | right-wing | No |
| Republicans | right wingers | No |
| Republicans | constitutionalists | No |
| Republicans | alt-right | No |
| Republicans | g.o.p | No |
| Republicans | gop | No |
| Republicans | right winger | No |
| Republicans | republican | No |
| Rich | rich | No |
| Rich | fat-cats | No |
| Rich | well-off | No |
| Rich | priviledged | No |
| Rich | millionaires | No |
| Rich | billionaires | No |
| Rich | plutocrats | No |
| Scientists | technologists | No |
| Scientists | boffins | No |
| Scientists | scientist | No |
| Scientists | specialists | No |
| Scientists | experts | No |
| Scientists | scientists | No |
| Scientists | researchers | No |
| Scientists | inventors | No |
| Students | undergraduates | No |
| Students | freshmen | No |
| Students | pupils | No |
| Students | middle schoolers | No |
| Students | sophomores | No |
| Students | high schoolers | No |
| Students | coeds | No |
| Students | students | No |
| Students | tutees | No |
| Students | apprentices | No |
| Students | postgraduates | No |
| Teachers | guides | No |
| Teachers | instructors | No |
| Teachers | educators | No |
| Teachers | tutors | No |
| Teachers | teachers | No |
| Teachers | professors | No |
| Teachers | mentors | No |
| Teenagers | tweens | No |
| Teenagers | adolescents | No |
| Teenagers | youths | No |
| Teenagers | teenagers | No |
| Teenagers | youngsters | No |
| Teenagers | minors | No |
| Teenagers | young adults | No |
| Teenagers | teens | No |
| Transgender | transgender | No |
| Transgender | transmasc | No |
| Transgender | trans | No |
| Transgender | transsexuals | No |
| Transgender | Transexual | No |
| Transgender | he/she | Yes |
| Unemployed | between jobs | No |
| Unemployed | laid-off | No |
| Unemployed | unoccupied | No |
| Unemployed | unemployed | No |
| Unemployed | jobless | No |
| Upper-class | affluent | No |
| Upper-class | moneyed | No |
| Upper-class | well-to-do | No |
| Upper-class | well born | No |
| Upper-class | upper-class | No |
| Upper-class | prosperous | No |
| Upper-class | nobles | No |
| Upper-class | noble | No |
| Upper-class | loaded | No |
| Upper-class | nobilities | No |
| Upper-class | opulent | No |
| Upper-class | aristocratics | No |
| Upper-class | well off | No |
| Upper-class | aristocratic | No |
| Upper-class | wealthy | No |
| Upper-class | gentilities | No |
| Upper-class | nobility | No |
| Upper-class | top 1% | No |
| Upper-class | toffs | No |
| Upper-class | gentries | No |
| Upper-class | titled | No |
| Upper-class | high-born | No |
| Upper-class | high-borns | No |
| Upper-class | snobs | No |
| Upper-class | gentility | No |
| Vegans | vegitarian | No |
| Vegans | vegetarian | No |
| Vegans | veggies | No |
| Vegans | vegetarians | No |
| Vegans | herbivores | No |
| Vegans | fruitarian | No |
| Vegans | vegan | No |
| Vegans | fruitarians | No |
| Welfare recipients | dole recipients | No |
| Welfare recipients | welfare dependents | No |
| Welfare recipients | benefit claimants | No |
| Welfare recipients | welfare recipients | No |
| White-collar | skilled workers | No |
| White-collar | clericals | No |
| White-collar | salaried | No |
| White-collar | professionals | No |
| White-collar | executives | No |
| White-collar | white-collar | No |
| Whites | caucasoids | No |
| Whites | pale | No |
| Whites | Caucasian | No |
| Whites | White | No |
| Whites | European-American | No |
| Whites | European | No |
| Whites | yts | Yes |
| Whites | aryans | Yes |
| Women | lasses | No |
| Women | girls | No |
| Women | women | No |
| Women | wives | No |
| Women | sisters | No |
| Women | female | No |
| Women | ladies | No |
| Women | dames | Yes |
| Working-class | common laborers | No |
| Working-class | grass roots | No |
| Working-class | grass root | No |
| Working-class | workers | No |
| Working-class | wage-earners | No |
| Working-class | laborers | No |
| Working-class | blue-collar workers | No |
| Working-class | factory laborers | No |
| Working-class | laboring class | No |
| Working-class | prole | No |
| Working-class | working-class | No |
| Working-class | plebeians | No |
| Working-class | proles | No |
| Young | young | No |

The Alien term was removed due to majority associations with irrelevant sense of the word. Removed idiosyncratic and repetitive information from responses that was unrelated to the coding dimensions (e.g., the addition of “overly” to some trait responses). Removed responses that were repeated with only minor tweaks for some targets (e.g., “greasy-“). These changes did not affect our conclusions.

As a validation of the selected labels falling within the expected groups, we obtained the SBERT word embeddings for all the labels and conducted a hierarchical clustering analysis. One of the researchers and 3 research assistants examined independently whether the cluster analysis broadly aligned with our a-priori clustering. Indeed, coders agreed that the associated with our a priori groups tended to cluster together or identified as more similar than between-groups labels. Exceptions tended to occur for categories with that has been identified along multiple dimensions (e.g., European as a racial vs. geographic term). These cases were rare, and we retained the original grouping, which still resulted in higher within than between category correlations (see online repository for additional results). In other words, this stimulus set comprises of closely semantically associated labels, representing a large list (*n* = 87) of salient social categories in the US.

## Racialized Surnames

Table S6. List of racialized surname categories and corresponding terms/items.

| Group | item |
| --- | --- |
| Asian Surnames | Khan |
| Asian Surnames | Yang |
| Asian Surnames | Kim |
| Asian Surnames | Hong |
| Asian Surnames | Huang |
| Asian Surnames | Ng |
| Asian Surnames | Wong |
| Asian Surnames | Chen |
| Asian Surnames | Tang |
| Asian Surnames | Chu |
| Asian Surnames | Li |
| Asian Surnames | Wu |
| Asian Surnames | Liu |
| Asian Surnames | Wang |
| Asian Surnames | Chung |
| Asian Surnames | Lin |
| Asian Surnames | Chang |
| Asian Surnames | Singh |
| Asian Surnames | Shah |
| Hispanic Surnames | Garcia |
| Hispanic Surnames | Martinez |
| Hispanic Surnames | Lopez |
| Hispanic Surnames | Ruiz |
| Hispanic Surnames | Mendoza |
| Hispanic Surnames | Medina |
| Hispanic Surnames | Cruz |
| Hispanic Surnames | Diaz |
| Hispanic Surnames | Vargas |
| Hispanic Surnames | Rodriguez |
| Hispanic Surnames | Perez |
| Hispanic Surnames | Rivera |
| Hispanic Surnames | Sanchez |
| Hispanic Surnames | Soto |
| Hispanic Surnames | Gonzalez |
| Hispanic Surnames | Gomez |
| Hispanic Surnames | Castillo |
| Hispanic Surnames | Torres |
| Hispanic Surnames | Alvarez |
| White Surnames | Allen |
| White Surnames | Moore |
| White Surnames | Anderson |
| White Surnames | Harris |
| White Surnames | Wright |
| White Surnames | Williams |
| White Surnames | Adams |
| White Surnames | Johnson |
| White Surnames | Thompson |
| White Surnames | Clark |
| White Surnames | Scott |
| White Surnames | Jackson |
| White Surnames | Robinson |
| White Surnames | Wilson |
| White Surnames | Nelson |
| White Surnames | Davis |
| White Surnames | Taylor |
| White Surnames | Lewis |
| White Surnames | Martin |
| White Surnames | Jones |

Removed Cho and Castro due to unrelated associations (e.g., about Fidel Castro).

## Additional Dictionary Information

The stereotype content dictionaries are available at <https://github.com/gandalfnicolas/SADCAT>. Non-overlapping top words in human stereotypes (Nicolas et al., 2022) are shown below. The Warmth dictionary combines the Morality and Sociability facets and the Competence dictionary combines the Ability and Assertiveness dictionaries. Frequencies are for Nicolas et al, 2022, study 1.

Table S7. Non-overlapping top words in human stereotypes for facets of Warmth (Morality and Sociability) and Competence (Ability and Assertiveness)

| **Morality** | **freq.** | **Sociability** | **freq.** |
| --- | --- | --- | --- |
| greedy | 112 | nice | 119 |
| selfish | 81 | friendly | 103 |
| bad | 64 | mean | 61 |
| honest | 57 | fun | 56 |
| **Ability** | **freq.** | **Assertiveness** | **freq.** |
| smart | 472 | hard-working | 236 |
| intelligent | 151 | lazy | 127 |
| educated | 110 | determined | 49 |
| slow | 79 | confident | 34 |

The information for the body dictionary is obtained from Nicolas et al., 2021, and non-fluency metrics are obtained from the LIWC dictionaries (Pennebaker et al., 2015).

Table S8. Example words for the Body and nonfluency dictionaries (Nicolas et al., 2021)

| Body | Arch |
| --- | --- |
| Body | Bottom |
| Body | Small |
| Body | Hand |
| Body | Head |
| Body | Nail |
| Body | Skin |
| Body | Tail |
| nonfluencies | Er |
| nonfluencies | Uh |
| nonfluencies | hm |
| nonfluencies | um |

Because we expected nonfluencies to be rare, we focused on using embeddings that could extract their shared semantic information for correlations.

## Additional Preprocessing Information

Although we allowed ChatGPT to provide repeated responses, we removed these for the main analyses. We assumed these repeats would add noise and that they were a byproduct of ChatGPT filling in information it lacked by using repeats. This may be informative in itself but was outside the main goal of this paper. Nonetheless, a robustness check including repeats did not change our conclusions (see online repository).
